# Reply to Hu et al.: Applying different evaluation standards to humans vs. Large Language Models overestimates AI performance

**DOI:** 10.1073/pnas.2406752121

**Published:** 2024-08-26

**Authors:** Evelina Leivada, Fritz Günther, Vittoria Dentella

**Affiliations:** ^a^Departament de Filologia Catalana, Universitat Autònoma de Barcelona, Barcelona 08193, Spain; ^b^Institut für Psychologie, Humboldt Universität zu Berlin, Berlin 10099, Germany; ^c^Departament d'Estudis Anglesos i Alemanys, Universitat Rovira i Virgili, Tarragona 43002, Spain

Dentella et al. (DGL) argued that 3 Large Language Models (LLMs) perform almost at chance in grammaticality judgment tasks, while revealing an absence of response stability (1). Hu et al.’s (HEA) “re-evaluation” led to different conclusions (2). HEA argue that i) “LLMs align with human judgments on key grammatical constructions,” ii) LLMs show “human-like grammatical generalization capabilities,” while iii) grammaticality judgments (GJs) are not the best evaluation method because they “systematically underestimate” these capabilities. While HEA’s aim to elucidate the abilities of LLMs is laudable, their claims are fraught with interpretative difficulties.

First, HEA replace the original task with minimal-pair probability measurements. Thus, they replace absolute judgments for individual sentences with relative judgments for pairs of sentences. To obtain the pairs, HEA doubled the DGL sentences but also added new ones. The result is that less than 50% of the input HEA used comes from DGL. While HEA frame this as a “re-evaluation,” they use both different materials and methodology; and several issues exist regarding the latter. Although grammaticality is a debated notion, whether a sentence violates a grammatical rule or not is not a matter of degree that requires a comparison of different sentences to be adjudicated (3). HEA argue that minimal-pair probability measurements (sum surprisal) are superior to GJs, but one needs to compare apples with apples. This is important because it affects HEA’s results: They argue that a minimal-pair analysis shows “at- or near-ceiling performance” for LLMs, yet when we consider the absolute sum surprisal^*^ of individual sentences rather than the relative sum surprisal of pairs, a different picture emerges. If surprisal is informative of grammaticality, it should be possible to set a surprisal threshold (ST) to tell apart grammatical from ungrammatical sentences. This is not what we find when we reanalyze HEA’s dataset; instead, we observe a massive overlap between the distributions (Fig. 1; accuracy 0.58 to 0.6). This is virtually identical to the overall accuracies of 0.59 and 0.56 reported in DGL, and far from at-ceiling. Consequently, the difference DGL found in humans vs. LLMs still holds.

**Fig. 1.**
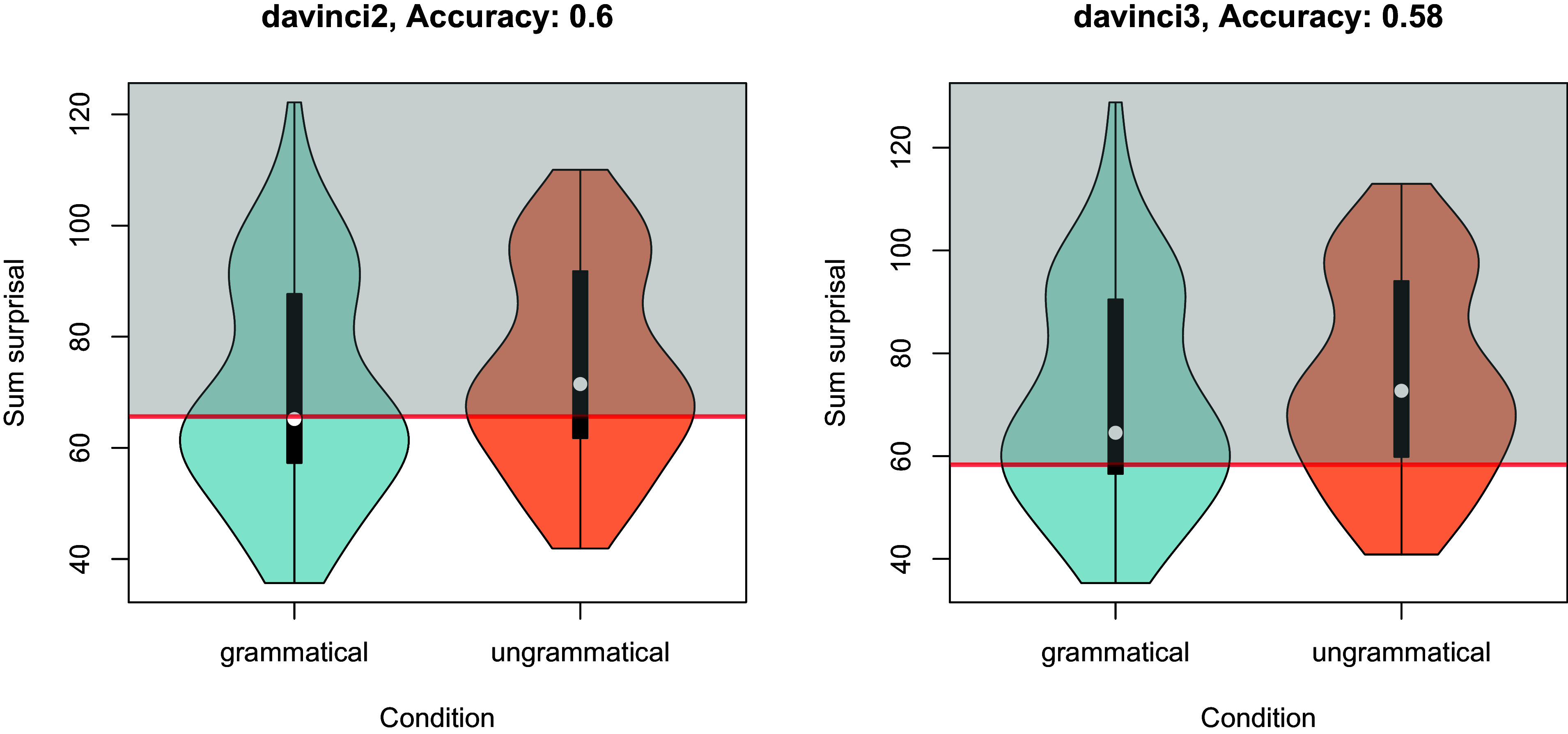
Distributions of sum surprisal in davinci2 and davinci3, for grammatical and ungrammatical sentences. The surprisal threshold that results in the classification with the highest accuracy is indicated by the horizontal red line. Any sentence with a sum surprisal higher than or equal to that threshold (in the gray area above the line) is classified as “ungrammatical,” while any sentence with a sum surprisal lower than that threshold is classified as “grammatical.” Even with an optimal ST that results in the highest possible classification accuracy, this accuracy is only at 0.60 for davinci2 and 0.58 for davinci3.

Second, HEA offer no human data to support the claim that LLMs show human-like capabilities. They took the DGL human data from GJs and compared them to the probability measurements of LLMs. By lumping the two types of findings together, HEA contradict themselves since, in previous work, Hu & Levy explain why this is not a good idea: “judgments elicited [(from LLMs)] through prompting are not the same as quantities directly derived from model representations” (4).

A third problem concerns unorthodox coding. HEA find that GPT-3.5/-4 outperform humans in GJs. This looks convincing, until one examines their prompting dataset. The LLMs should reply C (prompt=correct) or N (=incorrect). They replied, “I will be meeting with John and Karen C,” which HEA coded as target/accurate. Despite the alleged alignment with humans, it is hard to imagine that a human would give this response, and anyone would count it as target task behavior (5).

Holding LLMs accountable to a different standard of performance from humans, while attributing them human-like abilities, raises concerns (5) about the standards of testing the field wants to adopt in the road to ethical AI.

## Footnotes

^*^We observe the same results if we consider average surprisal or maximum surprisal instead of sum surprisal.
